# Commentary: How We Know What Not To Think

**DOI:** 10.3389/fpsyg.2020.00306

**Published:** 2020-02-25

**Authors:** Pablo Ezequiel Flores-Kanter

**Affiliations:** Research Department, University Siglo 21, Córdoba, Argentina

**Keywords:** memory, emotion, emotion regulation, stress, affect (emotion, mood, personality)

In a recent paper in TiCS, Phillips et al. (2019) reviewed and proposed a model that identifies a default sampling process in the way that people reason for alternative possibilities. In this shared mental adaptive sampling, two situational variables enter into consideration: Situational Probability and, Situational Value. Nevertheless, I think the model can be improved if the role of affect in cognitive judgments is taken into account.

For doing so, it is essential to answer: How can affective variables influence the selection of elements of our autobiographical memory, determining the subsequent judgments-thoughts that persons elaborated? Several psychological-affective models have been formulated to answer this question (Martin and Clore, 2001). One prominent and evidence-based theory was the Bower Network Theory of Affect (Singer and Salovey, 1988). In this associative network model, it is proposed that concepts, events, and affects can be represented as nodes within a network. A particular affective state (e.g., Distressed) can be described as a node interconnected with other nodes that represent, for example, phenomenological and physiological characteristics, as well as memories related to that affective-state. Based on this network model, it is possible to predict the probability of activation of specific mnesic components in function of the affective-state experienced at the moment (Flores Kanter et al., 2015). This network model proposes that affective-state can cause a change in the activation of mnesic categories in autobiographic memory, which in turn can influence cognitive judgments through a summation of activation effect (Forgas, 2000). So, when an affective state is activated, the affective-node propagates the activation to the rest of the nodes with which it is connected, including nodes representing episodic memories associated previously to the activated affect. This propagation of activation, as well as the activation of specific memories, would explain the effect of affective-state on thoughts or cognitive judgments.

There is recent evidence where it has been possible to verify this congruent-mood effect (positive and negative affective valence) in the development of various cognitive judgments, such as self-concept (Flores Kanter et al., 2015), self-efficacy (Medrano et al., 2016), and hopelessness-optimism judgments (Flores-Kanter et al., 2019). Even so, actual developments in affective neuroscience allow verifying this affective infusion effect at the brain structure's functional connectivity level. Synthetically, these works highlight the importance of differentiating between brain functioning in stress situations and recovery or homeostasis situations. These *Temporal Dynamics of Emotions and Stress* and the *Dynamic Functional Connectivity approach* (McNaughton, 2019) makes it possible to predict the differential role of bottom-up and top-down processes in the regulation of the cognitive-affective response, as well as the influence that these variables may have on memory.

For example, concerning the brain regions involved in affect regulation and stress response, the ascending signals from limbic subcortical structures (e.g., hypothalamus and amygdala) influence the activity of higher cortex structures through their connections to the medial prefrontal cortex (mPFC) (Weis et al., 2019). It is this activation that triggers self-referential repetitive negative thinking and is the basis for prolonged processing of negative information in the working memory (WM) (Stout et al., 2018). This two-way connection between limbic structures such as the amygdala and mPFC results in intensification and prolongation of the negative emotional response in its affective and expressive components (Park et al., 2019). This process would involve two primary networks. One, (a) the connections of the amygdala with the hippocampus and the caudate and putamen nucleus. This brain network explains the bias toward the harmful components of events produced in the memory (in both the formation and recovery of memories). Another, (b) activation of the right dorsolateral prefrontal cortex (right dlPFC), which plays a central role in anticipating the negative emotional stimulus, directing attention and cognitive resources toward it.

It is also essential to consider that other basic psychological processes can alter or interact with the described affective infusion mechanism^1^. Attention is one of these relevant processes, and there are recent experimental researches about attentional and emotional-distracts interactions that show some interesting results. For example, when attentional resources are limited (i.e., high level of perceptual load), the emotional-distractor processing is eliminated or limited (Gupta et al., 2016). Even so, from these experimental results, it can be seen that positive and negative emotions can interact with attention differently (Gupta, 2016, 2019). In this regard, it has been verified applying an inattentional blindness paradigm that in the high-load conditions, the percentage of participants with correct identification for the sad face was significantly worse than for the happy face (Gupta and Srinivasan, 2015). It has also been shown that the post-error slowing (i.e., PES) it been incrementing solely by a specific response-incongruent and task-irrelevant emotional positive cue (i.e., a happy face; Gupta and Deák, 2015). Therefore, the attentional process can moderate in a very complex manner the effect of a given stimulus on the subsequent emotional-cognitive response. Summarizing, these moderations depend on the interrelationship between the type of stimulus the person is facing (emotional-salient^2^ vs. neutral stimuli), and the attentional resources or attentional load that the person has at the time.

In conclusion, I believe that the relevant model proposed by Phillips et al. (2019) could be enriched by including (a) individual-transient variables (e.g., attentional resources/attentional load); (b) contextual variables (e.g., stress vs. safety situations; positive vs. negative emotional stimuli or neutral vs. salient stimuli); (c) the particular affective-cognitive responses that can be triggered in each case (e.g., bottom-up vs. top-down), in function of both the (a) individual and (b) the contextual variables; and (d) the specifics effect that these affective-cognitive response processes can have on cognition (e.g., in the moment judgment elaboration).

## Author Contributions

The author confirms being the sole contributor of this work and has approved it for publication.

### Conflict of Interest

The author declares that the research was conducted in the absence of any commercial or financial relationships that could be construed as a potential conflict of interest.

## Footnotes

^1^Other relevant moderate factors have been extensively discussed in the Affective Infusion Model (Forgas, 2000), but their consideration extends the objective of the present review.

^2^In the case of emotional-salient stimulus, the valence (positive vs. negative) is also relevant.
